# Changing liver utilization and discard rates in clinical transplantation in the ex-vivo machine preservation era

**DOI:** 10.3389/fmedt.2023.1079003

**Published:** 2023-02-23

**Authors:** Yara Azizieh, Lauren P. Westhaver, David Badrudin, Jeanette E. Boudreau, Boris L. Gala-Lopez

**Affiliations:** ^1^ Department of Pathology, Dalhousie University, Halifax, NS, Canada; ^2^ Department of Surgery, Université de Montréal, Montréal, QC, Canada; ^3^ Department of Microbiology and Immunology, Dalhousie University, Halifax, NS, Canada; ^4^ Beatrice Hunter Cancer Research Institute, Halifax, NS, Canada; ^5^ Department of Surgery, Dalhousie University, Halifax, NS, Canada

**Keywords:** machine preservation, organ discard, liver utilization, organ allocation, waitlist mortality

## Abstract

Liver transplantation is a well-established treatment for many with end-stage liver disease. Unfortunately, the increasing organ demand has surpassed the donor supply, and approximately 30% of patients die while waiting for a suitable liver. Clinicians are often forced to consider livers of inferior quality to increase organ donation rates, but ultimately, many of those organs end up being discarded. Extensive testing in experimental animals and humans has shown that ex-vivo machine preservation allows for a more objective characterization of the graft outside the body, with particular benefit for suboptimal organs. This review focuses on the history of the implementation of ex-vivo liver machine preservation and how its enactment may modify our current concept of organ acceptability. We provide a brief overview of the major drivers of organ discard (age, ischemia time, steatosis, etc.) and how this technology may ultimately revert such a trend. We also discuss future directions for this technology, including the identification of new markers of injury and repair and the opportunity for other ex-vivo regenerative therapies. Finally, we discuss the value of this technology, considering current and future donor characteristics in the North American population that may result in a significant organ discard.

## Introduction

More than 10,000 liver transplants (LT) are performed every year worldwide (1, 2). Despite the extensive use of live donation, the gap between listed recipients and available organs has widened over the years. Currently, the LT waitlist has increased by 8% annually in the UK, and the percentage of recipients on the waitlist in the United States will also rise over the coming years (3). To broaden the pool of available organs, grafts are increasingly used from extended criteria donors (ECD) and donors after circulatory deaths (DCD). Initially known as suboptimal or marginal livers, these organs usually come from donors of advanced age with significant steatosis, prolonged warm or cold ischemia, or major associated comorbidities (4). Ischemia-reperfusion injury (IRI) is inevitable during the transplantation of even healthy livers. Still, it is especially problematic when using organs from ECD because they are more vulnerable to ischemic insults (5, 6). As a result, they are often associated with poor graft function and reduced graft survival (1, 2).

ECD organs have also been associated with increasing discard rates (7). Given their poorer reported outcomes and the inability to safely predict their immediate function, many of these organs are discarded based on subjective analysis. Discards may occur early during the initial offer based on the donor's clinical history or later during the retrieval surgery upon macroscopic inspection due to hepatic or extrahepatic findings. Liver steatosis is by far the most frequent reason for discard, accounting for approximately 40%–60% of all non-used livers. This is particularly prevalent in western countries where non-alcoholic fatty liver disease (NAFLD) is becoming an epidemic as the population ages and diabetes and obesity are more prevalent (8). Previous studies have demonstrated that a high percentage of macrosteatosis is correlated with primary non-function (PNF), early allograft dysfunction (EAD) and even acute rejection (9).

Refined organ-recipient selection and new surgical strategies have allowed the safe use of ECD organs, with outcomes comparable to those of standard criteria donors (SCD) (4). One of the most significant advances came from *ex vivo* machine preservation (MP) of organs before transplantation. An old technology recently implemented into clinical settings (10). MP has proven to be safe in transplantation, with clear advantages for ECD grafts by allowing real-time assessment of the organ and by improving its primary function (10). This technology has been clinically validated in its two major modalities: hypothermic and normothermic preservation. It has provided a novel benchmark to objectively evaluate graft function using evolving metrics and markers that predict the success of an eventual transplant (11). Studies are now focused on finding ways to safely treat those organs while on MP using targeted strategies to ameliorate the ischemic injury, reduce pre-existing damage, and promote engraftment. MP has also been used to deliver high-dose therapeutics in the case of grafts with Hepatitis C Virus infection and to defat steatotic livers to allow transplantation, which would be otherwise toxic for recipients (1).

Our motivation to perform this literature search was the changing scenario for liver discard in times when MP is increasingly becoming available at various transplant centers. This paper was not intended to be a systematic review. We based our analysis on the assumption that *ex vivo* machine perfusion may be the key to improving ECD livers, expanding the donor pool and decreasing the discard rate of liver grafts (5, 12, 13). Here, we review the features of current and nascent methods of liver preservation during transplantation. We review the impacts of preservation methods on recipient outcomes and markers of liver health. Focusing on MP, we explore the effects of modalities on outcomes and discuss how this technology has contributed to modifying our acceptance and organ allocation criteria and their role in decreasing the discard of grafts.

## Current donor landscape and discard rates

### ECD/SCD/DCD

The discard rate of liver grafts in the United States has considerably increased from less than 1% in 1998 to approximately 6% in 2012, with a peak of roughly 6.5% between 2006 and 2009 (14). A similar analysis was performed in the United Kingdom for a 16-month period between 2016 and 2018, which revealed a total of 185 discarded liver grafts from causes including steatosis, warm ischemia time, cancer, fibrosis, poor machine perfusion and severe organ damage (15). Traditionally, the SCD for liver transplantation or the “ideal donor” must be of neurological determination of death (NDD), less than 60 years of age, with no history of viral or alcoholic hepatitis, no fat infiltration or tumours, no significant comorbidities including hypertension, and donating a whole good quality graft (16). Between 2003 and 2016, there was a total of 65,316 liver grafts procured from NDD in the United States who had died from head trauma, anoxia, stroke, and other non-specified reasons, 6,454 (9.88%) of the grafts were discarded (17).

On the other hand, ECD includes donors with advanced age, steatosis, prolonged ischemia time, viral hepatitis, and hypertension (16). As expected, due to the lower quality of ECD livers, the percentage of discarded livers is approximately threefold higher than that of SCD livers in the United States (18). A unique subset includes those livers from DCD, with the critical feature of the added primary warm ischemia that can result in unfavourable graft function, increased risk of ischemic cholangiopathy and re-transplant (4). The discard rate in this group has been observed to have increased by 24% since inception, which is significantly higher when compared to the SCD group, which has remained relatively stable at around 10% (17, 19).

### Advanced age

The average donor age for liver grafts has been increasing over the years because of the expansion of life expectancy and to combat the urgent unmet gap of available livers and recipients in need of transplantation (20, 21). However, grafts from older donors are still seen with reserve as these are often associated with increased biliary complications and graft failure, especially in recipients with hepatitis C (22). A concern from the use of older grafts is that with aging comes a decrease in liver regenerating capabilities resulting from decreased cell cycle and increased apoptosis and autophagy (22). Furthermore, hepatocytes tend to fall in numbers with older grafts, leading to the risk of early allograft dysfunction and primary non-function (23). When advanced donor age is combined with a DCD modality, ischemic damage negatively impacts post-transplant outcomes. Therefore, older donors should be carefully selected, and cold ischemia time (CIT) should be reduced whenever possible (20, 24).

Using donors 60 years and older has become more common, although they were once considered marginal (23). A study conducted on patient survival rates from donors 60 years of age or older at 1, 3 and 5 years post-transplant (86.8%, 72.6% and 67.6%, respectively) vs. donors younger than 60 years old (87.1%, 81.8%, and 75.5% respectively), revealed comparable results, indicating that donor age alone does not dictate post-transplant outcomes (25). Schlegel and collaborators reported that between 2005 and 2015, the median donor age at the University Hospitals Birmingham went from 28 to 68 years and that advanced age alone did not decrease recipient survival (26). This group also reported that grafts over 60 did not risk graft loss despite being DCD (26).

Although graft loss has decreased and patient survival from advanced donor age has increased over the years, the initial liver discard rate for these grafts has not (27). A study conducted between 2003 and 2016 included 4,127 grafts from donors over 70, where 747 were discarded, increasing from 11.6% to 15.4% (27). These findings suggest that older grafts should be utilized more often as age alone is not a predictor for adverse outcomes (25, 28). However, new strategies will be needed to decrease organ discard in countries where population aging is a reality and a challenge for the immediate future.

### Steatosis

Liver steatosis is the accumulation of lipids within hepatocytes and can occur due to metabolic disorders, obesity, old age, and alcoholism (6). There are two types of hepatic steatosis: macrovesicular and microvesicular steatosis. In macrovesicular steatosis, the nucleus is placed peripherally in the hepatocyte, and large fat vacuoles occupy most of the cytoplasm. In microvesicular steatosis the fat vacuoles are smaller (6). The most common liver disease affecting 17%–46% of the population is NAFLD, which causes insulin resistance, hypertension and dyslipidemia (29, 30). NAFLD is also accompanied by ceramide, a cytotoxic accumulation of lipids which promotes insulin resistance, apoptosis, and inflammation (29). It has also been found that patients suffering from NAFLD are also likely to have non-alcoholic steatohepatitis (NASH), leading to liver fibrosis and cirrhosis (30).

Steatosis is known for affecting liver regenerative responses and reducing tolerance to ischemic insult. Livers with macrovesicular steatosis are more susceptible to the damage resulting from the donation and cold preservation process, with mounting injury as preservation time increases (31). Primary non-function was the main negative post-transplant effect when using moderately and severely steatotic donor grafts, and these patients also experienced higher short-term mortality (32). As such, livers with steatosis are frequently discarded during the first organ offer or at the time of procurement, accounting for more than half of all discarded livers (8). Nonetheless, when livers are adequately selected, properly preserved, and paired with reasonable recipients, outcomes may be comparable to non-steatotic grafts (33).

### Donor risk index

The Donor Risk Index (DRI) was first published in 2006 to predict the survival of transplanted grafts after liver transplantation using seven donor characteristics that increase the risk of graft failure (14). These seven variables include age greater than 40 years, DCD donation, split grafts, race, height, cerebrovascular accident, and other causes of brain death (34). However, over the years, DRI has been considered a poor predictor and of little clinical benefit since the subjective assessments completed by surgeons are still the most powerful driver for organ acceptance or discard (35). In 2018, a new index called the discard risk index (DSRI) was introduced to grade grafts accurately solely based on the risk factors present when the graft is being offered to decide whether the graft was going to be discarded (36). This new index uses 15 predictors, including donor age, donor race, donor height, cause of death, DCD, chronic liver disease, gender, hepatitis B, hepatitis C, history of diabetes, history of hypertension, AST, ALT, total bilirubin, serum sodium and BMI (36). This is a validated scoring system, and its implementation can help in removing subjectivity from the organ assessment and combat the discard rate, especially in the more sub-optimal grafts (36).

## Preserving suboptimal organs

### Ischemia reperfusion injury (IRI)

Traditionally, there is an interval between organ removal from the donor and its implantation in the recipient. During this time, the liver experiences two stages of injury: ischemia (the stopping of blood flow), followed by reperfusion, when the blood flow is restored in the recipient. Damage resulting from these two events has been defined as ischemia-reperfusion injury (IRI), characterized by multiple cell signals leading to inflammatory response and ultimately associated with various degrees of graft dysfunction (5, 6). IRI is triggered by a fall in blood flow to the liver, decreasing oxygen availability, and depletion of ATP. This affects both endothelial cells and the extracellular matrix and can lead to apoptosis of hepatocytes resulting in liver necrosis (6, 37). Reactive oxygen species are generated upon reperfusion, which triggers an inflammatory response proportional to the preservation time and the quality of the underlying liver parenchyma (6). IRI is more significant in ECD livers, including DCD, as these organs are less suited to tolerate the resulting damage and are more associated with unfavourable immediate and long-term outcomes. The severity of IRI injury on the transplanted graft accounts for approximately 10% of early graft failure and 83% of re-transplantations, causing systemic inflammatory response syndrome or multi-organ failure in some cases (37, 38). Therefore, most efforts are aimed at decreasing the deleterious effect of IRI, but approximately 60% of DCD livers are still discarded due to IRI-related damage (39).

### Preservation solutions

The decline in donated liver health can be slowed by cooling the organ and reducing metabolic activity (40). Belzer and Southard pioneered the clinical implementation of a successful method of static cold storage (SCS) that is still used in most transplant centers. They demonstrated that livers could be better preserved by combining anaerobic hypothermic ischemia with a solution to ameliorate cell swelling and acidosis (41). Organ preservation solutions are used to minimize liver injury during anaerobic hypothermic ischemia by stabilizing temperature, hepatic structure, vasculature, osmolarity and pH while providing an opportunity to flush the organ from blood containing mediators of inflammation. During SCS with preservation solutions, the metabolic activity is reduced 10-fold, resulting in the arrest of the mitochondrial energy cycle (40, 41). Belzer and Southard developed the first preservation solution to be successful in clinical liver transplantation in the late 1980 s. The University of Wisconsin (UW) Solution was implemented through many challenges but ultimately demonstrated that solid organs could be safely preserved cold for a limited time, minimizing the impact of IRI (42, 43). Other formulations of preservation solutions have been validated in the last decades with similar performance, such as histidine-tryptophan-ketoglutarate (HTK), Celsior (CS), and Institut George Lopez (IGL-1).

HTK, a histidine buffer with tryptophan and ketoglutarate, has a very low viscosity that allows for faster cooling and is postulated to protect against biliary complications compared to UW (42, 44). CS is similar to UW and HTK but differs in that CS's buffer systems and substrate have high sodium and low potassium content, which limits calcium overload in the liver graft (42). Since CS also has a low viscosity in addition to its high sodium, low potassium, and antioxidant properties, so it seems ideal for preserving liver grafts (42). Finally, IGL-1 combines a cationic inversion and replacement of hydroxyethyl starch with polyethylene glycol, which could decrease IRI due to the improvement of hepatic microcirculatory changes (42).

There is no consensus on which preservation fluid is most effective in liver transplant, and their advantages may be context specific. UW and CS seem superior to HTK for primary non-function and biliary complications, respectively (42). But all alternate solutions to UW (HTK, CS. And IGL-1) demonstrated safety and efficacy for the preservation of deceased donor livers (42) and no significant differences in patient or graft survival (45). A subsequent study, however, demonstrated that HTK had the best efficacy for decreasing the primary dysfunction rate, biliary complications, and ICU stay time (46). CS was also associated with reducing rejection and early transplantation failure rates while increasing patient and graft survival rates (46). The preservation solution, IGL-1, in static cold storage is advantageous for suboptimal livers in transplantation (29).

### Prolonged ischemia time

Although SCS is the gold-standard method for preserving liver grafts before transplantation, it is limited by time, as organs can only be safely kept for up to 12 h (47). Prolonged cold ischemia amplifies IRI resulting in tissue damage that may be irreversible. Cold ischemia time is an essential factor in transplantation success, as prolonged times cause allograft damage, decreased ATP levels, increased free radical production, the release of cytokines, cellular dysfunction and apoptosis of the graft (Figure 2) (48). This mainly affects vulnerable organs such as those with steatosis, advanced donor age and DCD modality, where there is an increased risk of graft dysfunction, biliary strictures, and graft rejection after transplantation (6, 37). Typically, a CIT of over 12 h is predictive of a higher risk of graft loss, as it is the second most frequent cause of organ failure (12, 49). Prolonged CIT is also associated with higher posttransplant hospitalization and elevated serum bilirubin levels (49). Although there is no official time frame for CIT to stay within, most studies point to 12 h as a safe threshold. Still, this safety limit can be heavily influenced by patient risk and other factors that may arise during surgery (48). A study on the effects of CIT on hepatic allograft function concluded that although CIT did not cause any significant differences in histology or transfusional demand, it did cause elevation of serum transaminases and bilirubin, suggesting ischemic injury (48). For every hour CIT is prolonged, there is, on average, a 3.4% increase in the risk of graft loss (50). Even though all transplant centers strive to minimize CIT, geography and sometimes logistic obstacles prolonging the preservation times are often a cause for discard (50, 51). In countries like Canada and the United States with vast land extensions, CIT becomes the sole reason for declining perfectly usable organs when the donor and recipient centers are on opposite sides of the country, with long flight times involved.

Warm ischemia time (WIT) is defined as the ischemic insult of cells and tissue under normothermic conditions (i.e., body temperature or room temperature) (52). An hour of warm ischemia has been reported to result in reversible liver cell injury; however, 120–180 min of warm ischemia will provoke irreversible cell damage (6). WIT has been divided as primary (WIT1), referring to the period from the cessation of circulation in the donor until the organ is flushed with cold preservation solution and cooled topically until they reach a core temperature around 4°C. The secondary warm ischemia time (WIT2) is the period from removing the organ from the cold storage until it is reconnected to the recipient's circulation. Acceptable WIT2 in liver transplant ranges from 30 to 45 min, and longer times have been associated with increased graft loss at a 1.04 hazard ratio for every 10-minute increase (53). Prolonged WIT1 is a more impactful variable in deciding whether to accept or discard organs. WIT1 is particularly relevant in DCD settings where there is a waiting period from the withdrawal of life support to the actual cold flush of organs. Several studies have demonstrated a significant risk of PNF for periods longer than 30 min in DCD donors, with increasing interest in the functional WIT1 when there is a substantial deterioration of organ flow and oxygenation in the absence of asystole (54, 55). Prolonged WIT is hence the most crucial reason to discard livers from DCD donors.

## Machine preservation

*Ex vivo* preservation techniques were initiated at the beginning of the 20th century when Dr. Alexis Carrel and Charles Lindbergh employed perfusions in animal organs. This concept was later revisited by Belzer, who first successfully preserved a human kidney (56). However, the development of preservation solutions occurred in parallel, and when SCS became clinically feasible, it diminished enthusiasm for further developments in the machine perfusion (MP) field (56). Recent technological advances and a better understanding of liver physiology have allowed a resurgence of *ex vivo* MP in the last decade, with rapid worldwide clinical implementation (57, 58).

MP aims to maintain circulation through the liver before transplantation. Continuous circulation of preservation solutions allows for better diffusion into the microcirculation and hence, more homogenous graft protection (10). It also provides for the modification of the organ temperature depending on the preservation strategy and the administration of nutrient- and oxygen-rich fluids through the organ. Since this is a dynamic form of preservation, multiple parameters can be measured in real-time to monitor the organ's performance over time. Together with different markers of liver function, these factors provide a more accurate assessment of the organ's viability and its predicted function post-transplant, which permits a decision to accept or discard the organ based on more scientific evidence (59). Several approaches to MP are in use or development, and a key point of research is determining the temperature approach to maintain organ vitality *ex vivo*. Hypothermic, sub-normothermic, and normothermic preservation are the temperature modalities, and all seem to be beneficial in preserving SCD and ECD livers alike (60). Combining approaches, such as MP, with temperature variations also seem to provide advantages (6), with new experimental works aimed to extend preservation for several days (47).

### Hypothermic machine perfusion

Hypothermic machine perfusion (HMP) preserves livers between 4° and 12°C using the benefits of the anaerobic hypothermic ischemia concept paired with continuous perfusion throughout the microcirculation of the organ (61). HMP has been used clinically without oxygen supplementation, much like machine perfusion used for kidneys today and was found to be feasible in terms of their acceptability rate (43). Studies conducted by Guarrera et al. (62) and Dutkowski et al. (63) both demonstrated that the use of HMP on liver grafts before transplantation yielded exceptional acceptance rates (Table 1).

**Table 1:** Published discard and acceptance rates of liver grafts on MP vs. SCS.

Discard rate of liver grafts on MP		Acceptance rate of liver grafts on MP		Discard rate of liver grafts on SCS		Acceptance rate of liver grafts on SCS	
Source	Modality	Source	Modality	Source	Modality	Source	Modality
Bral et al., (59)	NMP: 1/10	Bral et al., (59)	NMP: 9/10	Bral et al., (59)	SCS: 0/30	Bral et al., (59)	SCS: 30/30
Bral et al., (64)	NMP: 3/46	Bral et al., (64)	NMP: 43/46	Dutkowski et al., (65)	SCS: 0/50	Dutkowski et al., (65)	SCS: 50/50
Dutkowski et al., (66)	HMP: 0/8	Dutkowski et al., (66)	HMP: 8/8	Dutkowski et al., (66)	SCS: 0/8	Dutkowski et al., (66)	SCS: 8/8
Guarrera et al., (62)	HMP: 2/20	Guarrera et al., (62)	HMP: 18/20	Guarrera et al., (62)	SCS: 2/20	Guarrera et al., (62)	SCS: 18/20
Mergental et al., (15)	NMP: 9/31	Mergental et al., (15)	NMP: 22/31	Guarrera et al., (67)	SCS: 0/30	Guarrera et al., (67)	SCS: 30/30
Mergental et al., (68)	NMP: 1/6	Mergental et al., (68)	NMP: 5/6	Henry et al., (69)	SCS: 0/15	Henry et al., (69)	SCS: 15/15
Nasralla et al., (70)	NMP: 16/137	Nasralla et al., (70)	NMP: 121/137	Nasralla et al., (70)	SCS: 32/133	Nasralla et al., (70)	SCS: 99/133
Ravikumar et al., (71)	NMP: 0/20	Ravikumar et al., (71)	NMP: 20/20	Ravikumar et al., (71)	SCS: 0/40	Ravikumar et al., (71)	SCS: 40/40
Reiling et al., (72)	NMP: 0/10	Reiling et al., (72)	NMP: 10/10	Selzner et al., (73)	SCS: 0/30	Selzner et al., (73)	SCS: 30/30
Van Rijn et al., (74)	DHOPE: 0/10	Van Rijn et al., (74)	DHOPE: 10/10	Van Rijn et al., (74)	SCS: 0/20	Van Rijn et al., (74)	SCS: 20/20
Vogel et al., (75)	NMP: 11/11	Vogel et al., (75)	NMP: 0/11	Van Rijn et al., (76)	SCS: 4/78	Van Rijn et al., (76)	SCS: 74/78

This is the most accessible modality to clinically implement as it maintains a concept like the widely accepted SCS with minimal requirements in terms of training, supervision, and cost. It also demonstrated to be an effective method to preserve initially discarded livers with superior outcomes regarding EAD, biliary complications, and patient survival compared to traditional SCS (67). HMP also allows CIT extension for up to 20 h safely, without significant complications after transplant (12).

New evidence is now supporting better preservation during HMP when combined with oxygenation. Hypothermic oxygenated machine perfusion (HOPE) (77). HOPE provides energy to the hypoxic liver by removing metabolic waste while preventing the exhaustion of ATP and mitochondrial edema (78). It also reduces secondary damage during reperfusion by inhibiting the activation of endothelial cells and leukocytes, further increasing the success of the graft (78). HOPE can be conducted by exclusive perfusion of the portal vein or by perfusion of both the portal vein and hepatic artery, also known as dual-hypothermic oxygenated machine perfusion (DHOPE) (60). Studies conducted on HOPE have shown that this technology is safe and feasible and can improve post-transplant outcomes, especially in liver grafts from DCD donors (77, 79). This technology also seems to reduce the incidence of EAD (79), and it improves the 1-year graft survival rate when compared to SCS (79). Even though HMP is not widely used clinically, new trials are underway to make this technology available to more transplant physicians, with the potential to significantly contribute to reducing discard rates.

### Sub-normothermic machine perfusion

Sub-normothermic machine perfusion (SNMP) is another alternative in liver transplantation where organs are dynamically preserved between 20° and 34°C (80, 81). SNMP remains considerably unexplored, but it has equally demonstrated significant benefits in liver preservation, mainly in experimental settings (82). In addition to the protection of hepatocellular mitochondria, microcirculation was also found to be spared from damage in SNMP, compared to cold ischemia, where tissues swell, leading to a higher risk of reperfusion failure (82). One of the most hazardous damage-associated molecular patterns known as HMGB-1, a known marker of tissue injury, was significantly suppressed in SNMP compared to SCS (82). Interestingly, SNMP seems particularly beneficial for steatotic livers as these grafts generate higher levels of ATP and maintain more integrity during SNMP, compared to normothermic preservation (83). Hence, there is a theoretical opportunity to reduce the discard of severely steatotic livers through SNMP. Still, the actual clinical implementation of this modality is to be seen.

### Normothermic machine perfusion

Normothermic machine perfusion (NMP) is the most popular and widely used form of MP because it mimics the physiological characteristics of the liver. NMP maintains the graft by providing oxygen, nutrients and other vasoactive drugs at a temperature of 37° (84). This results in a better energetic balance for liver cells resulting in fewer mediators of IRI (85). Since the first clinical report came out of Oxford University in 2016 (71), multiple clinical studies have demonstrated that NMP is also feasible, safe, and able to improve liver preservation, resulting in less EAD and shorter hospital stays (59, 73). These studies also demonstrated that NMP could be used to safely expand preservation time in ECD and DCD livers for up to 24 h to allow for a more detailed characterization of the graft without compromising outcomes (59). Furthermore, seminal large multi-center confirmed the initial findings and demonstrated the reproducibility of results in two different NMP systems in European centers (70) and, more recently, in various centers in the United States (86).

**Figure 1:**
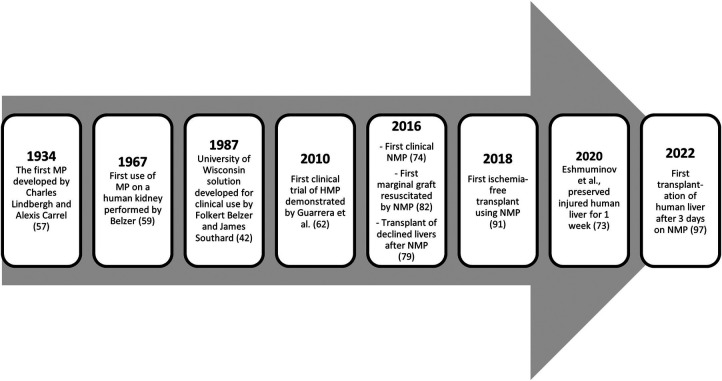
Important landmarks of MP throughout history.

New studies now report that NMP can increase the acceptance rate of liver grafts, including those traditionally discarded (15, 59, 64, 68, 71, 72, 87) (Table 1). The first human liver transplantation using a marginal allograft resuscitated with NMP (Figure 1) demonstrated that after 422 min of cold ischemia, NMP could establish hepatic arterial flow, portal venous flow, perfusate blood gases, and bile production (88). A recent paper further supported this observation by Mergental and collaborators, reporting the successful transplantation of 22 out of 31 livers previously discarded by all programs in the United Kingdom after being tested and perfused with NMP (15).

### Markers for graft functions

Ideally, a biomarker for graft health in liver transplantation should predict how much injury the organ has sustained before transplantation and predict the risk of EAD and PNF. Currently, there is no single biomarker with such characteristics, and instead, clinicians and scientists use a combination of indicators before donation and during preservation (11). Today, the most studied biomarkers for graft function during MP are those traditionally used in the clinical assessment of liver function (89). Early work in clinical MP found that the injury detected after transplantation could also be seen during preservation, as aspartate aminotransferase (AST) and alanine aminotransferase (ALT) serum levels on MP correlated with the AST and ALT levels in the recipient after transplantation (62). Transaminase levels in machine perfusate are associated with the organ's quality and have been used to help decide the acceptance of the graft (90, 91).

Although liver transaminases are widely used in the posttransplant evaluation of the graft, the interpretation of its values during all types of MP may be challenging, as AST and ALT seem to accumulate during the perfusion period without necessarily indicating graft dysfunction (92). Instead, other indicators of liver health, such as lactate clearance, pH of the perfusate, bile production, vascular flows, and pressures, have been used along transaminase levels to accurately assess the viability of the organ and the quality of preservation (85). Multiple efforts are made to identify more accurate molecular indicators of organ health, and various candidates in the field of proteomics, metabolomics and genomics are now under study. MicroRNAs (miRNA) are small noncoding RNAs present in many biological processes. miRNA have also been identified during liver IRI, miRNA-122 being the most highly expressed in hepatocytes, particularly during ischemic injury (11). miRNA-122 has been extensively studied during clinical liver injury and is now being tested in MP settings, with preliminary association to injury and subsequent EAD (93). Inflammatory cytokines and chemokines are also targeted as liver health/injury/recovery markers. These are well-known markers during liver transplant, and their value has also been explored during MP. Guarrera and collaborators observed a significant increase of ICAM-1, IL-8 and TNF-α during HMP, associated with liver IRI (94). More recent data focuses on the role of mitochondria during liver preservation. Since this microorganelle actively participates in IRI and recovery from it, multiple potential biomarkers are under study. ATP production has been reported as a direct indicator of liver health during MP (95, 96). Our group is currently investigating the value of circulating mitochondrial DNA in MP perfusate as an indicator of organ health and a predictor of post-transplant graft function. Despite the exciting discoveries in biomarkers during *ex vivo* machine preservation, many of these are usually hampered by the low clinical uptake, given the prolonged processing time to measure them, the accessibility across transplant centers, and their cost (97).

## MP flattening the discard curve

Because machine perfusion is known to reduce IRI and allow the option to deliver targeted therapeutics directly to the graft, it is theoretically our best option to reduce the liver discard rate (13). Several studies have demonstrated that MP is associated with a lower discard rate of liver grafts compared to the gold standard SCS. Most notably, a randomized trial conducted by Nasralla and collaborators revealed the discard rate in SCS to be significantly higher (24.1%) compared to that of NMP which (11.7%) (70) (Table 1). Another recent controlled study evaluating NMP has also shown that organ discard decreases with access to NMP technology, as physicians are willing to assess grafts in real time before declining the organ (86). Other studies frequently report adequate liver viability when using NMP for organs that all active centers in a region have discarded. In most of these studies, organs show adequate function ex-vivo, and some have been transplanted into recipients without detrimental consequences (13, 15, 59, 64, 68, 71, 72, 87, 98). Our own group is currently performing a pan-Canadian study to evaluate the impact of this technology on waitlist mortality as an indirect indicator of graft acceptance.

Donation after cardiocirculatory death rapidly expands in all regions as an alternative to widen the donor pool. Despite the intrinsic risk of EAD and ischemic cholangiopathy, DCD is a sizable worldwide source for transplantation (54). A reason for the success of using these organs is the strict acceptance criteria built around these donors. However, these criteria may become restrictive in today's changing donor landscape, especially those referring to the WIT1 and the expected preservation time. Many centers are evaluating MP as a tool to expand these criteria based on existing observations that longer WIT1 is not necessarily associated with graft loss (12, 49). New research is now using NMP to avoid ischemia altogether, connecting the MP system directly to the donor and later to the recipient, transitioning the organ from one stage to another without any ischemic insult (99, 100). The first case of “ischemia-free” liver transplant (IFLT) was reported in 2017 (101), followed by a non-randomized trial including 38 patients receiving IFLT, compared to 130 patients receiving conventional transplantation. Only two recipients (5.3%) in the IFLT experienced EAD compared to 50% in the traditional group, with added benefits when using an ECD graft (102).

In liver steatosis, MP is also seen as a potential tool for defatting livers and decreasing discard (1). MP reduces intracellular lipids by improving liver metabolism and increasing the movement of intracellular triglycerides, which helps lower cellular injury and enhances microcirculation in the liver (1). Specifically, when NMP is used on steatotic livers for up to 6 h, the concentration of triglycerides decreases in addition to improved intracellular lipid metabolism (103). It is believed that hepatocyte triglyceride may drop as much as 38% after NMP (103). Further studies are looking to perform active defatting strategies (104, 105) and ultimately combine these interventions with longer perfusion times to allow for more efficient fat removal (85). Our group is currently working on novel technology to monitor hepatocyte triglyceride in real-time in a non-invasive fashion (106). The study was validated in animal models and is now advancing into clinical trials.

## Future criteria for acceptance/discard

**Figure 2:**
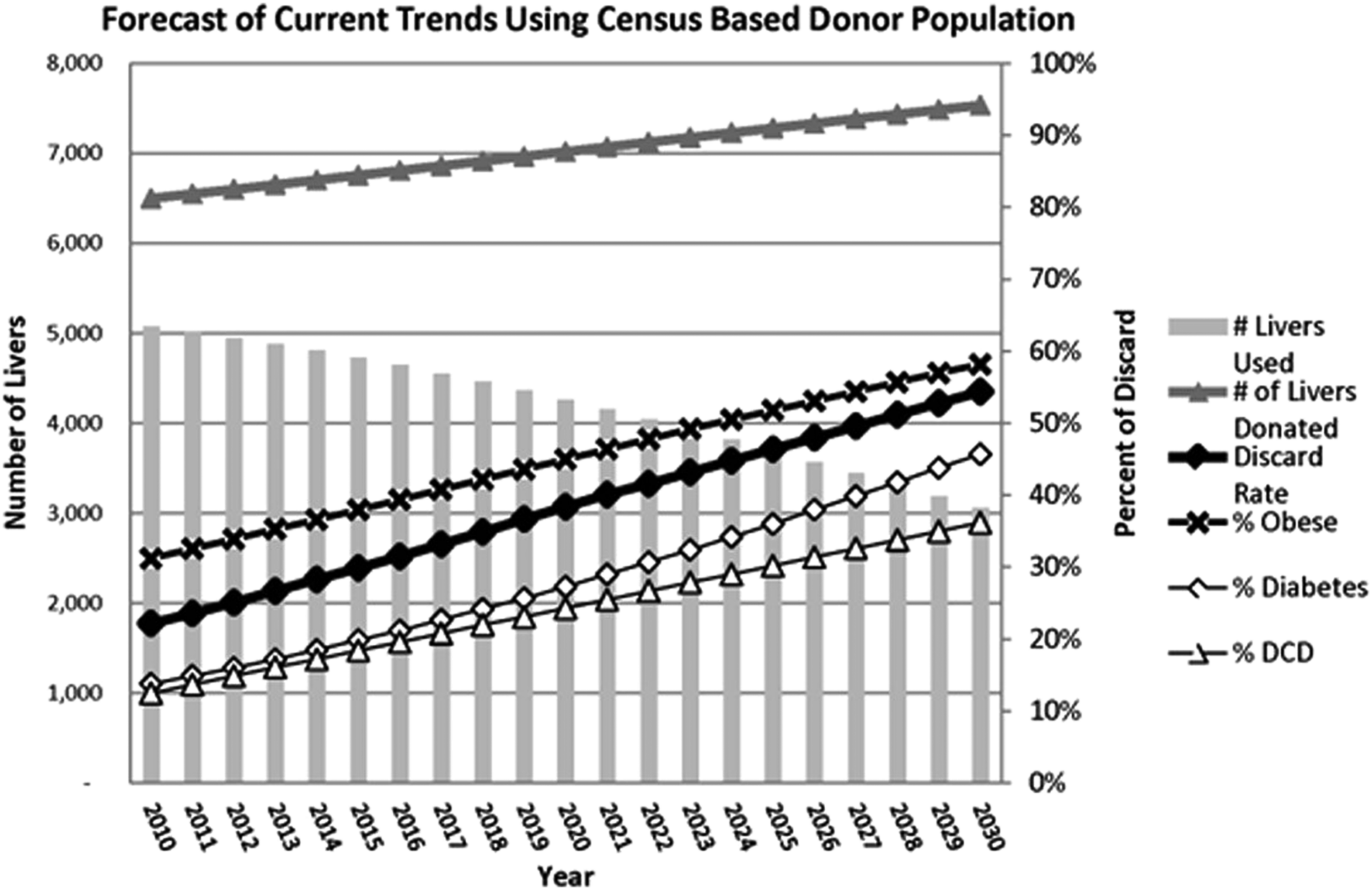
Statistical estimates provided by Orman E. et al., 2015 illustrating the decrease of LT in the United States by the year 2030 due to an increase of discarded grafts because of the increased incidence obesity, diabetes, and DCD. Reproduced with permission (8).

After many years of clinical transplantation, SCS seems sufficient for low-risk organs with short ischemia because normal livers are somewhat resilient against IRI and show adequate post-transplant outcomes (27, 28). However, the donor landscape is increasingly shifting towards using ECD to incorporate an older population, steatotic livers, DCD grafts, donors from medical assistance in dying, and more. This will inevitably lead to a high discard of livers if we continue using traditional techniques. Orman and collaborators conducted a population study based on United States data forecasting donor characteristics by 2030, revealing shocking numbers. The study stated that if the current donor utilization and practices do not change, there would be 2,230 fewer grafts for transplantation in the year 2030 due to the declining quality of donors, resulting in a discard rate close to 60% of all donated livers (Figure 2) (8). However, the authors also pointed out that through the increasing use of ex-vivo MP techniques, the discard curve could be bent as this technology can turn many unusable organs into safe alternatives for many patients on the wait list (8). It will be paramount to adequately understand the real impact of these unfavourable donor variables once NMP is widely available for use.

Future directions should focus on new research to identify more accurate biomarkers and predictors of liver health during preservation and regenerative therapies that can be applied during MP to rescue marginal livers. Another avenue for development is related to optimizing the technology to safely preserve livers for days, as recently demonstrated by the group at the University of Zurich (85, 107). This will permit more realistic rescue strategies and, eventually, organ exchange through more extensive networks. Only a comprehensive plan will maintain or even increase our current transplant rates.

## Conclusions

With an ever-increasing organ demand and limited supply of adequate donors, further improvements in the transplantation process, including strategies to minimize IRI, will be necessary to avail less-ideal organs for transplant. The expansion of the donor pool relies mainly on increasing the usability of ECD organs by bringing forward strategies to ameliorate ischemic injury or to improve those grafts deemed unusable safely. Machine preservation seems to be the ideal tool for this purpose by combining old concepts with new technology and discovery. Research rapidly shows its value by allowing comprehensive clinical implementation and innovation, quickly transferred from bench to bedside. The future of liver transplantation is inspiring, and traditional organ acceptability criteria will necessarily change in light of such disruptive technology.
